# Bending the boundaries: the many facets of endophilin-As from membrane dynamics to disease

**DOI:** 10.1007/s00018-025-05856-w

**Published:** 2025-09-23

**Authors:** Shiqiang Xu, Emilie Rigaux, Dorian Hène, Henri-François Renard, Louise Thines

**Affiliations:** 1 UNamur, Namur Research Institute in Life Sciences, Research Unit in Cell Biology, Rue de Bruxelles 61, B-5000 Namur, Belgium; 2 UCLouvain, Louvain Institute of Biomolecular Science and Technology, Group of Molecular Physiology, Croix du Sud 4-5, B-1348 Louvain-La-Neuve, Belgium

**Keywords:** Endophilin-A1/2/3, SH3GL2/1/3, EEN, Endocytosis, Trafficking, BAR domain, Autophagy, Receptor, Signaling, Cell adhesion, Neurodegeneration, Cancer, Migration, Proliferation

## Abstract

The endophilin-A proteins (EndoAs) are Bin/Amphiphysin/Rvs (BAR) domain proteins with key roles in both clathrin-mediated (CME) and clathrin-independent endocytosis (CIE). Humans have three differentially expressed EndoAs, EndoA1, -A2, and -A3, encoded by the *SH3GL2/1/3* genes, respectively. Their functions primarily arise from their N-terminal BAR domain, which senses and induces local membrane curvature, and C-terminal SH3 domain, which mediates interactions with various proline-rich domain-containing partners. Among others, EndoA-mediated endocytosis coordinates synaptic vesicle recycling, as well as internalization of cell adhesion molecules, ligand-stimulated receptors, and pathogens. Consequently, EndoAs influence key cellular processes like neurotransmission, signaling, cell adhesion, and infection. Importantly, EndoA dysregulation has been observed in several pathologies, notably neurodegeneration, cardiovascular diseases, and cancer. This review provides an overview of the function and regulation of the EndoA proteins in CME and CIE, and explores their lesser-characterized involvement in other processes such as autophagy. It further addresses how these functions contribute to physiological processes and the development of pathologies, with a particular focus on cancer pathophysiology. Together, it emphasizes non-redundant roles of EndoA proteins in various cellular processes and highlights the complex relationship between membrane trafficking and diseases.

## Introduction

Bin/Amphiphysin/Rvs (BAR) domain proteins play key roles in membrane dynamics by sensing and inducing local membrane curvature through their BAR domain [1]. Based on domain features, they are classified into classical BAR (including N-BAR), Fes/CIP4 homology (F)-BAR, and inverse (I)-BAR families [2]. Among the N-BAR proteins are endophilins. Mammals express five highly-similar endophilins: EndoA1, -A2, and -A3 (see Table 1 for corresponding genes and aliases), as well as EndoB1 and -B2. Though evolutionarily conserved, *Drosophila melanogaster* and *Caenorhabditis elegans* have only one EndoA and EndoB orthologs, suggesting gene duplication and differentiation throughout evolution. Given specific functions in recently identified endocytic modalities, combined with complex relevance in cancer and other diseases, this review focuses solely on the EndoA proteins.

**Table 1:** Overview of the human EndoAs: coding genes, protein aliases, and pathological implications

	Gene	Protein aliases	Cancer		Other pathologies
			Pro-tumoral	Tumor suppressor	
EndoA1	*SH3GL2*	EEN-B1; Endophilin-1; SH3 domain protein 2 A; SH3GL2; SH3p4	Gastric, esophageal, and brain cancer	Brain, urothelial, breast, vulvar, head and neck, lung, and eye cancer	Neurodegeneration: Alzheimer and Parkinson diseases, epilepsy
EndoA2	*SH3GL1*	EEN fusion partner of MLL; EEN; Endophilin-2; SH3 domain protein 2B; SH3GL1; SH3p8	Breast, bone, liver, colorectal, and brain cancers, lymphoma, leukemia	None reported	Cardiac injury and hypertrophy, atherosclerosis, proteinuria, kidney fibrosis, autoimmune diseases, antibody deficiencies, pathogen infection
EndoA3	*SH3GL3*	EEN-B2; Endophilin-3; SH3 domain protein 2 C; SH3GL3; SH3p13	Brain, myeloma, colon, and skin cancer	Brain, lung, and head and neck cancer	Huntington disease

Abbreviations: *EEN* extra eleven-nineteen, *MLL* mixed-lineage leukemia, *SH3GL* SH3 domain-containing GRB2-like

**Fig. 1:**
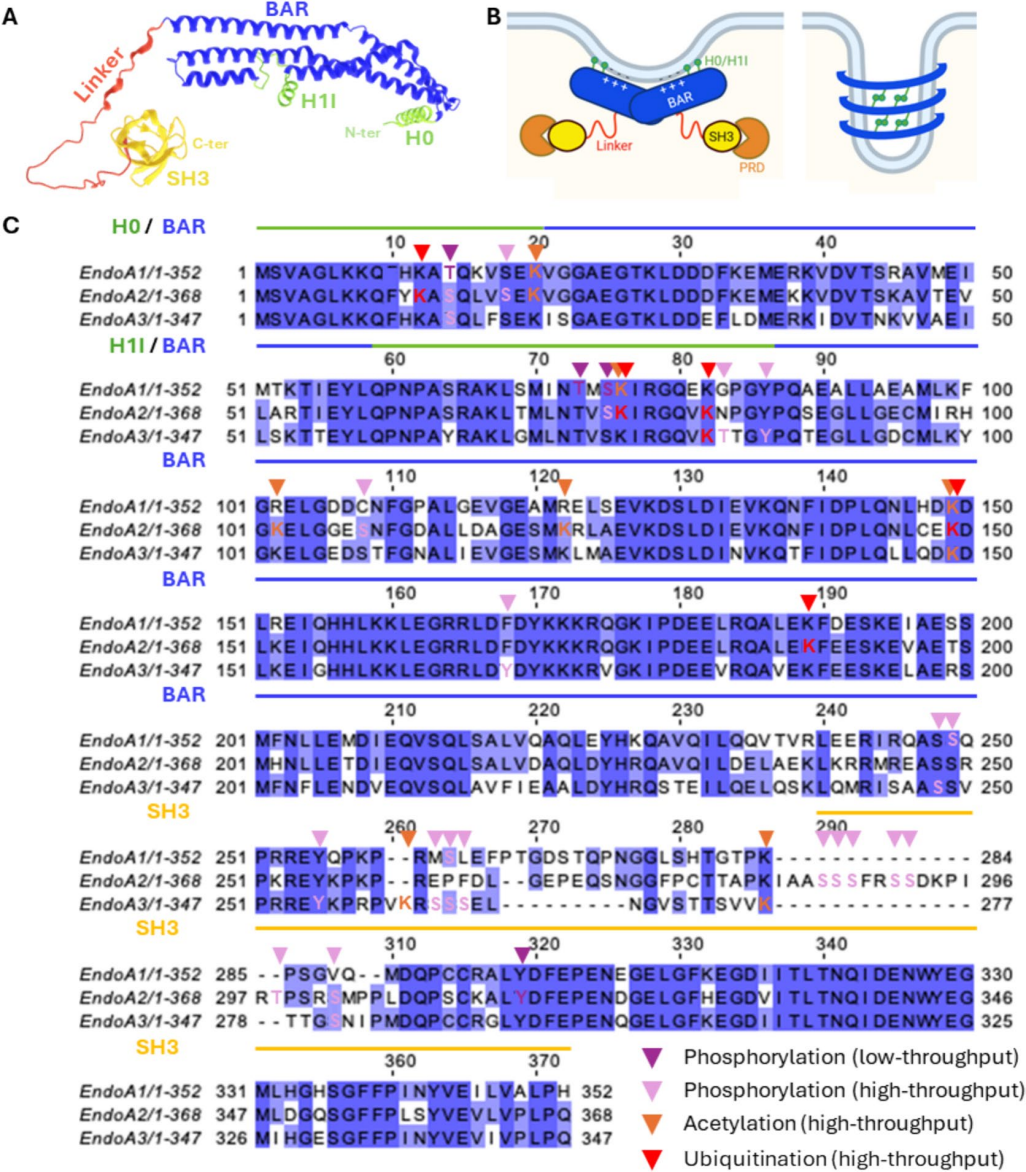
The endophilin-A (EndoA) proteins. **A** Predicted structure of EndoA3 from AlphaFold. The BAR domain (including H0 and H1I amphipathic helices) and SH3 domain are well predicted. In contrast, the linker is predicted with low probability, reflecting flexible, disordered features. Note that the BAR and SH3 domain structures have also been experimentally resolved (see https://www.rcsb.org/). **B** EndoAs commonly form dimers through their BAR domain, which scaffold biological membranes via electrostatic interactions. The H0 and H1I amphipathic helices insert into membranes to favor membrane binding/bending, whereas the SH3 domain binds proline-rich domain (PRD)-containing partners. EndoAs also associate into higher-order oligomers forming helical lattices, through anti-parallel association of their H0 helices, promoting membrane tubulation. **C** Alignment of the three human EndoAs, highlighting post-translational modifications identified in high- and low-throughput studies.

All EndoAs (~ 40–50 kDa) comprise an N-terminal N-BAR domain (249 amino acids) composed of three anti-parallel α-helices (H1-H3) that forms crescent-shaped, positively-curved dimers (Fig. 1A, B) [3]. Positively-charged residues exposed at the concave BAR domain surface interact with negatively-charged headgroups of phospholipids to scaffold and bend membranes [4, 5]. The N-BAR domain also comprises two ~ 20 residue-long amphipathic helices – H0 at their N-terminus and H1-insert (H1I) within the H1 helix – that insert into membranes to increase membrane interactions and, by displacing lipids, further promote membrane bending (Fig. 1A, B) [6–8]. However, such “wedging” effect remains debated and may depend on local lipid composition [9, 10]. Interestingly, shallow versus deep membrane insertion of H0 appears to regulate endophilin functions in vesiculation and tubulation, respectively [11, 12]. Higher-order EndoA oligomers can also form helicoidal lattices that scaffold membrane tubules. Such oligomers are stabilized by H0:H0 interactions from different dimers in neighboring helix turns (Fig. 1B) [13]. Importantly, although the endophilin N-terminal portion was initially reported to have lysophosphatidic acid acyl transferase activity promoting membrane curvature [14], such an activity is now refuted [15].

At the C-terminus, endophilins have an SH3 domain (60 residues) that forms a β-barrel with a hydrophobic groove for binding of proline-rich domain (PRD)-containing proteins (Fig. 1A, B) [16, 17]. Key endophilin-SH3 binding partners include the large GTPase dynamin, the phosphoinositide phosphatase synaptojanin [18–20], and the cytoskeleton regulator N-WASP [21, 22]. A flexible linker (37–58 amino acids), less conserved and less characterized, connects the N-BAR and SH3 domains (Fig. 1A-C). Chimeric EndoAs with swapped linkers reveal that this region influences EndoA endocytic functions [23]. The linker also harbors several post-translational modification (PTM) sites (Fig. 1C), as well as putative Ca^2+^-binding sites, further suggesting implications in regulation. In humans, EndoA1 is predominantly expressed in the brain, EndoA3 in the brain and testes, and EndoA2 is ubiquitously expressed [19, 24, 25]. Adding complexity, each EndoA has multiple splice variants (https://www.uniprot.org/).

## Neuronal EndoAs in synaptic vesicle and AMPA receptor trafficking

**Fig. 2:**
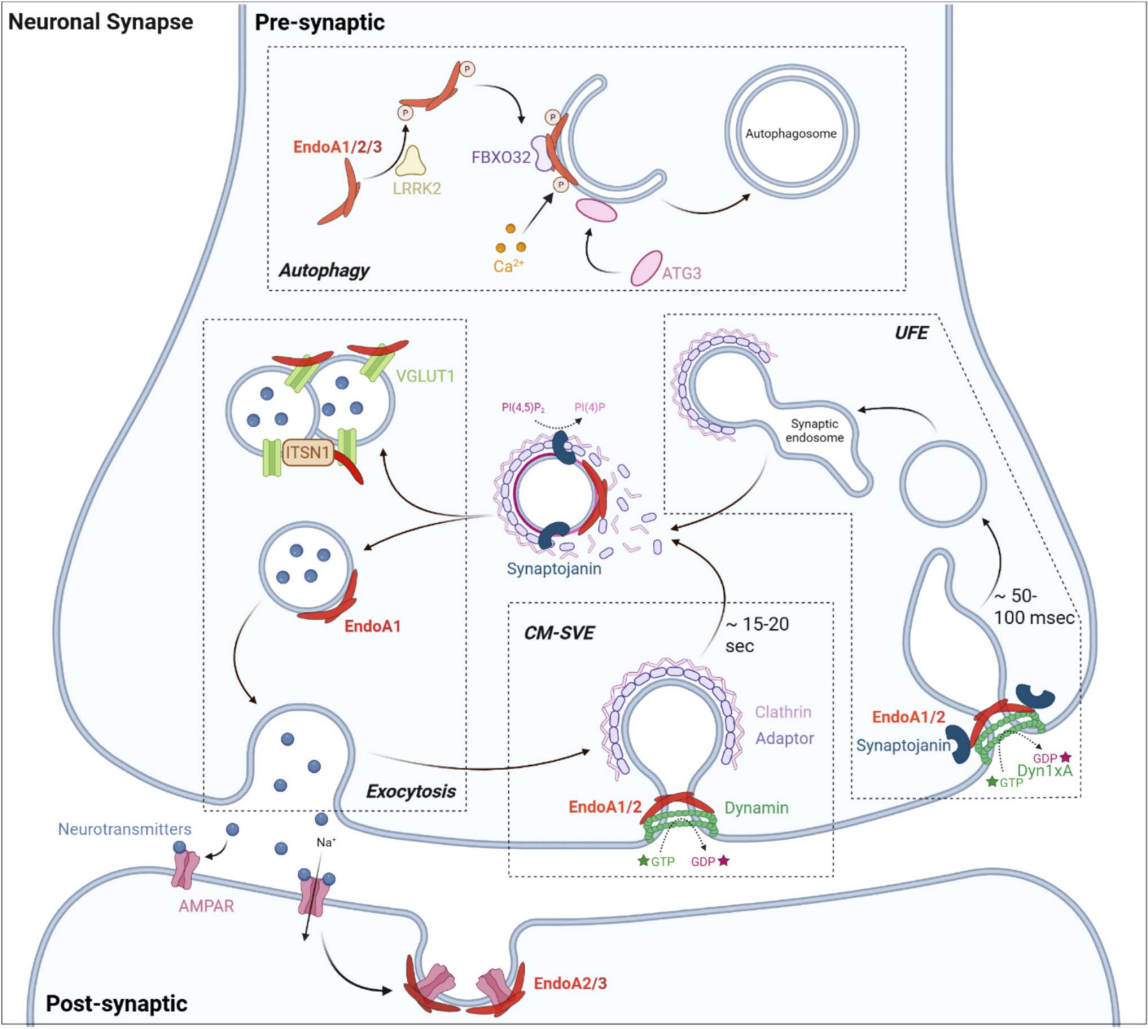
The multiple functions of neuronal EndoAs at synapses. In pre-synaptic neurons, EndoAs contribute to synaptic vesicle recycling via both clathrin-mediated synaptic vesicle endocytosis (CM-SVE) and clathrin-independent ultrafast endocytosis (UFE). Both processes require concerted actions with dynamin GTPase (or its variant Dyn1xA in UFE) and synaptojanin phosphatase. EndoA1 further stimulates synaptic vesicle exocytosis (modulated by VGLUT1 interactions and intersectin-1 (ITNS1)-induced vesicle clustering). Finally, all three EndoAs, together with Atg3 and FBXO32, operate in autophagosome formation (stimulated by LRRK2-mediated phosphorylation and Ca^2+^ signaling). In post-synaptic neurons, EndoA2 and EndoA3 are involved in AMPA receptor (AMPAR) endocytosis. Given these various contributions, EndoAs are essential for neuronal synapse homeostasis. Generated with BioRender.

EndoAs primarily associate with the plasma membrane where they participate in clathrin-mediated endocytosis (CME) (Table 2), as evidenced by their detection on clathrin-coated pits (CCPs) [20, 26–29]. In neurons, EndoAs play central functions in clathrin-mediated synaptic vesicle endocytosis (CM-SVE), which is essential for recycling membranes following exocytosis of neurotransmitter-filled vesicles (Fig. 2, Table 2) [30]. Consistently, *D. melanogaster, C. elegans,* and mouse mutants lacking EndoA show impaired SVE [26, 31–36]*.* During CM-SVE, EndoAs coordinate vesicle budding through BAR domain-mediated membrane scaffolding (Fig. 2) [11, 32, 33, 37, 38]. Supporting this, deletion of the BAR domain disrupts CM-SVE in *C. elegans*, while reconstitution of EndoA-null mutants with its BAR domain rescues this defect [33]. Similarly, EndoA1 trapping by antibody injection in lamprey synapses, as well as EndoA partial loss-of-function in *D. melanogaster* neurons, lead to an accumulation of shallow CCPs, reflecting impaired budding [32, 37, 38]. Other studies report that EndoAs operate in vesicle fission and clathrin uncoating via SH3 domain-mediated interactions [19, 39]. First, EndoAs interact with GTP-bound dynamin at CCP necks, promoting fission upon GTP hydrolysis [20, 40]. Subsequently, synaptojanin bound to EndoAs dephosphorylates PI(4,5)P_2_ to PI(4)P, causing clathrin coat disassembly (Fig. 2) [18, 26, 27, 35, 41]. Consistently, SH3 interfering peptides competitively binding to EndoA binding partners in lamprey synapses cause accumulation of both arrested CCPs (fission defects) and free clathrin-coated vesicles (uncoating defects) [20, 42]. Interestingly, EndoAs interact with other proteins during CM-SVE, including the N-BAR protein amphiphysin [43] and the RhoGAP oligophrenin-1 [44] for EndoA1, and voltage-gated Ca^2+^ channels for EndoA2 [45], highlighting that EndoAs mediate the assembly of functional endocytic machineries. Despite well-established roles in CM-SVE, the exact stages at which EndoAs are involved are still debated. For example, studies indicate that the BAR domain is sufficient during CM-SVE, suggesting that functions of EndoA-SH3 is dispensable in fission and uncoating [33]. Moreover, EndoA1-3 triple knockout newborn mice show no defects in vesicle scission [26], suggesting dispensable functions that can be compensated by other endocytic proteins. The specific contributions of each EndoA to CM-SVE also remains unclear: while double or triple EndoA knockout is required to observe SVE defects in mice [26], indicating partial redundancy, knockdowns in rat hippocampal neurons suggest that SVE is mostly sustained by EndoA1 and -A2 but, despite its high neuronal expression, not by EndoA3 [46].

**Table 2:** EndoA-mediated endocytic modalities

	CM-SVE	UFE	FEME	EndoA3-mediated
Stimulus	Synaptic activity		Receptor ligand stimulation/Pathogen binding	Galectins: clustering (promotion) or lattice trapping (inhibition)
Cargoes	Synaptic vesicles		Receptors (GPCRs, RTKs, IL-2, axon guidance, BCRs, AMPARs)/Pathogens (Shiga & Cholera toxins, *T. cruzi*, EV71)	Ig-like CAMs (ALCAM, L1CAM, ICAM1)
Main EndoA mediator(s)	EndoA1-2		EndoA2	EndoA3
EndoA roles (with main other actors)	Bending (clathrin) Scission (dynamin) Clathrin uncoating (Synaptojanin)	Scission (Dyn1xA, Synaptojanin) Clathrin uncoating (Synaptojanin)	Priming (Cdc42, FBP17, CIP4, SHIP1/2, Lpd) Carrier elongation, & fission (dynamin, cytoskeleton, dynein, Bin1)	Carrier elongation & fission (PSTPIP1, Rac1, cytoskeleton, myosin, kinesin)
Main cellular outcomes	Neurotransmission		Signaling/Infection	Cell adhesion & migration/Immune response

Abbreviations: *AMPAR* AMPA receptor, *BCR* B-cell receptor, *EV71* enterovirus71, *FEME* fast endophilin-mediated endocytosis, *GPCR* G protein-coupled receptor, *Ig-like CAM* immunoglobulin-like cell adhesion molecule, *IL-2* interleukin-2, *Lpd* lamellipodin, *RTK* receptor tyrosine kinase, *CM-SVE* clathrin-mediated synaptic vesicle endocytosis, *UFE* ultrafast endocytosis

Clathrin-mediated synaptic membrane retrieval occurs within 15–20 s [47, 48]. Importantly, neuronal EndoAs also mediate clathrin-independent ultrafast endocytosis (UFE), where synaptic membrane retrieval takes 50–100 ms only (Fig. 2, Table 2) [49–51]. UFE shares mechanistic similarities with CM-SVE: it is primarily driven by EndoA1 and -A2 [52], and involves interactions with dynamin and synaptojanin. Specifically, EndoA1-2 in complex with synaptojanin and the dynamin splice variant Dyn1xA are required to constrict the neck of UFE pits, facilitating fission (Fig. 2) [52, 53]. Accordingly, EndoA1-2 depletion, as well as disruption of their interaction with Dyn1xA, results in stalled UFE pits with wider necks [52, 53]. Although UFE is clathrin-independent, internalized vesicles subsequently fuse with synaptic endosomes, from which clathrin-coated vesicles bud to regenerate synaptic vesicles [50, 54]. Like in CM-SVE, EndoA1-2, together with synaptojanin, are involved in clathrin uncoating of such newly-budded synaptic vesicles (Fig. 2) [52, 55]. In line with this, *C. elegans* EndoA mutants accumulate synaptic endosomes, reflecting altered breakdown into new synaptic vesicles [55, 56].

Interestingly, EndoAs in pre-synaptic neurons also operate in synaptic vesicle exocytosis (Fig. 2): docking of EndoA1 dimers at the surface of exocytic vesicles stimulates their fusion with the plasma membrane, possibly by inducing membrane remodeling. In contrast, EndoA1 binding to the vesicular glutamate transporter 1 (VGLUT1) on synaptic vesicles limits their exocytosis, likely by restricting EndoA1 availability for its stimulating docking [57]. Moreover, EndoA1 and VGLUT1 form complexes with the adaptor protein intersectin-1, which induces synaptic vesicle clustering and reduces spontaneous exocytosis in the absence of stimulation [58]. EndoA1 and intersectin-1 also cluster vesicles near neurotransmitter release sites to enable fast activity-induced, EndoA1-dependent replenishment of those sites for sustained neurotransmission [59]. In neurosecretory cells, EndoA1 and EndoA2 interactions with intersectin-1 further promote priming and fusion of exocytic vesicles [60]. Finally, EndoAs also support exocytosis-endocytosis coupling in auditory hair cells by stimulating pre-synaptic Ca^2+^ influx by the Cav1.3 channel and interacting with the Ca^2+^ sensor otoferlin [61]. Importantly, EndoA functions in pre-synaptic exocytosis remain to be further validated: contradictory findings in hippocampal cells indicate that the EndoA1/2/3:VGLUT1 interaction does not influence synaptic vesicle exocytosis, but rather stimulates activity-driven endocytosis, thereby contributing to VGLUT1 recycling during synaptic stimulation [62].

EndoAs also function in post-synaptic neurons, where EndoA2 and -A3 mediate AMPA receptor (AMPAR) endocytosis through interaction with the cytoskeleton-associated protein Arc (Fig. 2) [63, 64]. EndoA3 further stimulates AMPAR endocytosis by binding and activating the Arf6 guanine nucleotide exchange factors (GEF) BRAG2a [65]. Together, these findings underscore the versatility of EndoAs in modulating synaptic vesicle and AMPA receptor trafficking at synapses.

## Functions in CME of cell surface receptors

Non-neuronal EndoAs also play a role in CME, particularly during dynamin-mediated membrane fission. In 3T3 fibroblasts, exogenously-expressed EndoA2 and dynamin are co-recruited to CCPs for vesicle scission [29, 66]. In the same cell line, overexpression of the EndoA1 SH3 domain inhibits membrane fission during CME of the transferrin receptor, likely by disrupting SH3-mediated interactions [67]. Similarly, EndoA1-3 triple depletion or EndoA2 overexpression in human melanoma cells reduces or enhances dynamin recruitment to the plasma membrane, respectively [68]. In kidney fibroblasts, overexpressed EndoA3 further facilitates membrane fission by interacting with N-WASP and promoting actin polymerization [21]. Interestingly, EndoA1-3 also accumulate at CCPs prior to fission to support the formation of elongated tubular necks [28], suggesting additional roles in CCP maturation. EndoAs in CME are documented to coordinate internalization of cell surface receptors. For example, EndoA1-3 bind to the adaptor CIN85, forming a complex recruited by the ubiquitin ligase Cbl to activated EGFR and c-MET receptor tyrosine kinases (RTKs), initiating their endocytosis [69, 70]. In HEK293T cells, EndoA2 overexpression and its binding to the BPGAP1 RhoGAP also stimulates EGF-stimulated EGFR endocytosis [71]. The three EndoAs further promote ligand-stimulated EGFR internalization in 3T3 fibroblasts by recruiting the cytoskeletal regulator lamellipodin to CCPs to stimulate actin polymerization [72]. EndoA1 and -A2 similarly bind the Arf6 GEF EFA6, promoting Arf6 activation and, subsequently, CME of the transferrin receptor [73]. In conclusion, despite the lack of a consensus mechanism, EndoAs appear to form endocytic complexes with various proteins to stimulate CME of multiple receptors across various cell types. It is however important to note that some of these studies, though referring to CME, did not clearly demonstrate the clathrin dependency of their modalities. Nevertheless, EndoA functions in CME appear to be partially conserved throughout evolution: the *Arabidopsis thaliana* homologs SH3P1, −2, and −3 promote clathrin uncoating through interactions with the auxilin-like vesicle uncoating factor and the SAC9 phosphoinositide phosphatase [74–77], and further regulate the trafficking of clathrin-coated vesicles via actin interactions [78].

While studies report EndoA functions at various stages of CME (including SVE), the dynamics and molecular drivers of their recruitment are still debated. EndoA1 has been proposed to be recruited to CCPs through direct interactions with intersectin-1 [79]. Additionally, dynamin depletion reduces EndoA recruitment to the plasma membrane, suggesting potential cooperative recruitment [68]. Adding controversy, several studies report that EndoAs may inhibit CME. For instance, EndoA3 overexpression in kidney fibroblasts impairs CME of transferrin and dopamine D2 receptors [23], while EndoA1 and -A3, by recruiting ataxin-2 to the plasma membrane, reduces EGFR CME [80]. Moreover, high levels of EndoA1, either on artificial membranes or in SK-MEL-2 cells, inhibits fission by inserting into dynamin helices [81, 82]. Other studies even suggest peripheral, dispensable roles for EndoAs in CME, as it was detected only in a subset of CCPs in NIH-3T3 cells [29] and EndoA1-3 triple knockdown in human melanoma cells does not affect transferrin uptake [68]. Collectively, these observations indicate complex, context-dependent functions of EndoAs in CME. Importantly, with most studies to date being carried out with ectopically expressed, tagged EndoAs or in *in vitro* systems, EndoA contributions to CME warrants further investigation in endogenous contexts.

## EndoA2-mediated CIE of ligand-stimulated receptors and pathogens

**Fig. 3:**
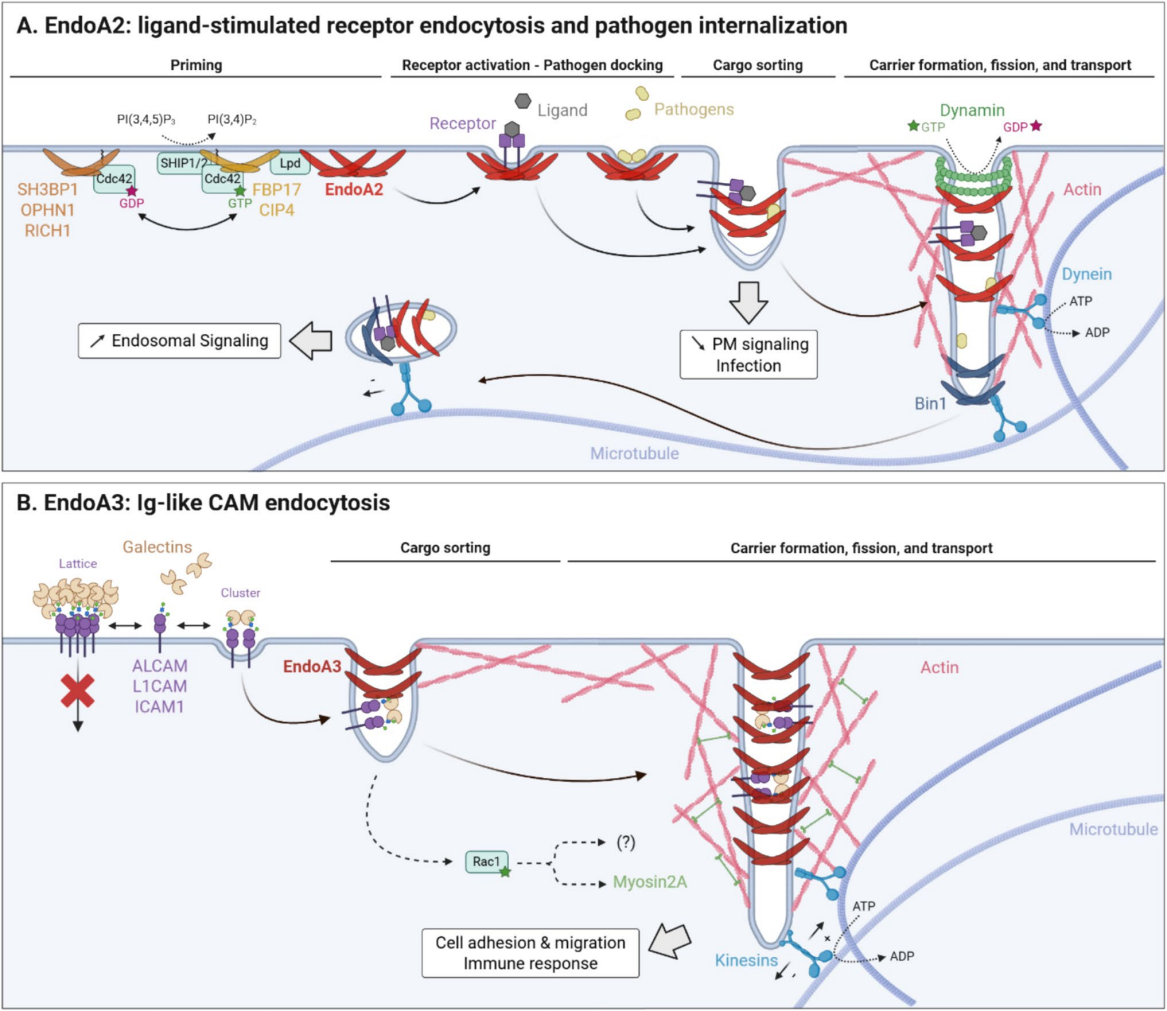
EndoA2- and EndoA3-clathrin-independent endocytosis. **A** EndoA2-mediated FEME of ligand-stimulated receptors is characterized by a priming step (involving Cdc42 GTPase, FBP17 and CIP4 BAR domain proteins, SHIP1/2 phosphatases, lamellipodin (Lpd) cytoskeletal regulator, and SH3BP1, OPHN1, and RICH1 GAPs) followed by, upon cargo capture, carrier formation, elongation, and fission through concerted actions with dynamin GTPase, Bin1 BAR domain protein, dynein molecular motor, and the cytoskeleton. Consequently, FEME influences signaling, reducing or increasing transduction from the plasma membrane or endosomes, respectively. Importantly, this mechanism is hijacked by several pathogens (bacterial toxins, viruses) to enter host cells, which is likely initiated upon EndoA2 recognition of initial membrane curvature induced by pathogen:receptor clustering. **B** EndoA3-mediated CIE of Ig-like CAMs is regulated by extracellular galectins, that either trap highly-glycosylated cargoes into lattices or stimulate their endocytosis upon clustering, depending on cargo glycosylation status and local galectin concentrations. While the cargo sorting mechanism remains elusive, carrier formation, elongation, and fission require the actions of Rac1 GTPase, myosin2A and kinesin molecular motors, and the cytoskeleton. Importantly, EndoA3-mediated internalization of Ig-like CAMs influences cellular adhesion, migration, and immune response. Panels A and B are based on [94] and [105], respectively. Generated with BioRender.

In addition to CME, EndoAs mediate clathrin-independent endocytic modalities, including fast endophilin-mediated endocytosis (FEME) (Fig. 3A, Table 2) [83]. Although initially broadly attributed to all EndoAs, FEME mostly relies on EndoA2 [83, 84]. FEME internalizes various ligand-stimulated receptors, including G protein-coupled receptors (GPCRs) (β1- and α2A-adrenergic, dopamine (D3/D4), and muscarinic acetylcholine 4 receptors), RTKs (EGF, HGF, VEGF, PDGF, NGF, IGF-1 receptors), cytokine (interleukin-2) receptors, axon guidance receptors (Plexin A1 and ROBO1), B-cell receptors (BCRs), and AMPARs [83, 85–88]. Importantly, FEME is also hijacked by the Shiga and Cholera toxins [89], the parasite *Trypanosoma cruzi *[90], and the enterovirus 71 [91] for host cell entry.

Mechanistically, FEME is characterized by a priming step, in which EndoA2 is constantly pre-clustered in discrete plasma membrane patches, likely facilitating rapid endocytosis upon cargo detection [92]. Priming involves GTP-Cdc42 recruiting the F-BAR proteins FBP17 and CIP4, which then recruit SHIP1/2 phosphatases. SHIP1/2 convert PI(3,4,5)P_3_ into PI(3,4)P_2_, triggering lamellipodin recruitment, which ultimately recruits EndoA2. The formation of EndoA2 priming patches may additionally involve liquid–liquid phase separation [93]. In the absence of nearby cargo, Cdc42 is deactivated by RICH1, SH3BP1, and Oligophrenin GTPase-activating proteins (GAPs), leading to patch disassembly [92, 94]. In contrast, upon cargo recognition, EndoA2 patches transition into tubular FEME carriers. Activated receptors are recognized by EndoA2 predominantly at the leading edge, either via direct binding to PRD-containing cytosolic tails or indirectly through adaptor proteins. Initial pit bending is driven by increased local EndoA2 concentration upon receptor capture, and/or ligand-induced receptor crowding [89, 94]. In the case of pathogens, binding to and clustering of their lipid or protein receptor likely induces local membrane curvature [95, 96], further recognized by cytosolic EndoA2 [89]. Subsequently, the N-BAR domain protein Bin1 recruits dynein molecular motors onto nascent carriers which, by moving along microtubules, promotes elongation and fission [87, 89, 97, 98]. The application of such pulling forces on tubular membranes where EndoA2 scaffolding generates frictional resistance to lipid diffusion favors tubule neck squeezing and scission, this mechanism being called friction-driven scission (FDS) [89, 98]. In cells, optimal scission additionally requires actin polymerization, supported by EndoA2 interactions with the actin regulators VASP, Mena, and NHSL1/2, as well as dynamin [89, 99, 100]. Interestingly, EndoA2 foci colocalize with the scaffold protein Alix [101], but also with other BAR domain proteins (ASAP1, SNX9, Pacsin2, and srGAP1), further suggesting cooperative actions that remain to be investigated [92].

## EndoA3-mediated CIE of immunoglobulin-like cell adhesion molecules

More recently, EndoA3 was identified to mediate endocytosis of ALCAM (Activated Leukocyte Cell Adhesion Molecule, also called CD166) [84], L1CAM (L1 Cell Adhesion Molecule, or CD171) [102], and ICAM1 (Intercellular Cell Adhesion Molecule 1, or CD54) (Fig. 3B, Table 2) [103]. EndoA3 colocalizes with these CAMs at the plasma membrane and is pulled down by the cytosolic tails of ALCAM and L1CAM [84, 102, 103], supporting its direct involvement in their endocytic uptake. All cargoes identified to date are immunoglobulin-like cell adhesion molecules (Ig-like CAMs) with heavily glycosylated extracellular portions, suggesting common EndoA3-dependent recognition patterns that remain to be elucidated. Interestingly, EndoA3-mediated CIE of ALCAM is stimulated by extracellular galectin-8, which likely clusters glycosylated cargoes and glycosphingolipids to initiate plasma membrane invagination [84], consistent with the glycolipid–lectin hypothesis [104]. In contrast, although galectin-1, −3, and −8 colocalize with L1CAM-positive endocytic structures, extracellular galectins inhibit L1CAM internalization, possibly due to the formation of galectin lattices trapping it at the membrane [102]. This suggests that galectins may differentially regulate EndoA3-mediated CIE, depending for example on cargo glycosylation patterns that influence galectin interaction affinities, or on local extracellular galectin concentrations. Inside the cell, EndoA3- and ALCAM-positive endocytic structures dynamically associate with the actin cytoskeleton and microtubules [105]. The current mechanistic model suggests that *(i)* Rac1-stimulated activity of the molecular motor myosin 2 A (and possibly of other Rac1 effectors) promotes actin remodeling and increases membrane tension to favor membrane deformations, and that *(ii)* kinesin motors pull nascent EndoA3-positive carriers along microtubules to favor tubule elongation and FDS [105]. L1CAM endocytosis also requires the F-BAR protein PSTPIP1 which, consistently with its larger intrinsic curvature radius, is recruited prior to EndoA3 [102], again supporting cooperative, sequential BAR domain protein actions during endocytosis. Following internalization, both ALCAM and ICAM1 undergo retromer-dependent retrograde transport and subsequent polarized redistribution to the plasma membrane [103], implying possible shared post-endocytic fates for EndoA3 cargoes.

## EndoA functions: beyond endocytosis?

Recent studies have expanded the functional repertoire of EndoA proteins beyond endocytosis, including in post-endocytic trafficking, as supported by their detection on endosomes [106]. For instance, EndoA2 binds to the microtubule-associated protein CRMP2, which facilitates the movement of AMPAR-positive endosomes along the cytoskeleton to enable receptor recycling [107]. Moreover, while EndoA1 is not required for endocytosis of the RTK TrkB, it is recruited by the endosomal protein retrolinkin to TrkB-positive early endosomes and directs sorting to late signaling endosomes, promoting downstream ERK signaling [106, 108]. In line with this, and as mostly observed in cancer cell lines (see below), EndoA-mediated trafficking of ligand-stimulated receptors influences intracellular signaling, decreasing signal transduction from the plasma membrane or increasing signal propagation from endosomes. Unfortunately, the molecular determinants governing distinct signaling outcomes (e.g., reduced or enhanced signaling from the plasma membrane or endosomes, activation of selected downstream pathways) following endocytosis remain poorly understood. Curiously, EndoAs may also have peripheral, endocytosis-independent functions in signaling, as EndoA1 was reported to interact with the Germinal Center Kinase-like Kinase, leading to JNK kinases activation [109].

EndoAs also participate in protein turnover and autophagy. In *Drosophila* neurons, EndoA promotes the formation of highly curved cytosolic membranes to which the autophagy factor Atg3 can dock to initiate autophagosome formation [110]. In mammalian neurons, all three EndoAs also interact with the E3 ubiquitin ligase FBXO32 to cooperatively promote autophagosome biogenesis and regulate protein turnover (Fig. 2) [111]. Similarly, in bovine aortic smooth muscle cells, EndoA2 stimulates ubiquitination and autophagic degradation of the Ca^2+^-activated Cl^−^ channel TMEM16A [112]. More indirectly, EndoA2 from rat cardiomyocytes promotes autophagy by augmenting EndoB1 interaction with the autophagy regulator Beclin-1 [113]. Importantly, and as discussed later, EndoA functions in autophagy are regulated by phosphorylation [110] and Ca^2+^ signaling [114]. In addition, the three EndoAs contribute to apoptosis by binding Alix, promoting cytoplasmic vacuolization [115]. Moreover, EndoAs localize to actin-rich protrusions such as dendritic spines [116] and podosomes [117], and may facilitate their morphogenesis. Indeed, EndoA1 interacts with the adaptor protein p140Cap in dendritic spines to promote actin remodeling essential for spine formation [116, 118]. Intriguingly, EndoA2 is also detected in the nucleus of hematopoietic, fibroblast, and epithelial cells, where it is hypothesized to undergo nucleo-cytoplasmic shuttling during cell cycle. In *A. thaliana,* during cytokinesis, SH3P2 in complex with dynamin-related protein 1 A induces tubulation of *trans*-Golgi-derived vesicles to form planar cell plates, further suggesting EndoA functions in cell cycle [119]. In summary, while EndoA functions are clearly not restricted to endocytosis, future studies are required to further delineate their mechanisms of action in such diverse cellular processes.

## Regulation of the EndoA proteins

Being implicated in endocytosis and other key cellular processes, EndoAs must be tightly regulated to modulate their dynamic recruitment to the plasma membrane or other cellular structures upon given signals. PTMs, including phosphorylation, acetylation, and ubiquitination, have been identified on all three EndoAs in high-throughput mass spectrometry screens (Fig. 1C) (https://www.phosphosite.org/). EndoA1 is phosphorylated by leucine-rich repeat kinase 2 (LRRK2) at Thr^73^ and Ser^75^ within the H1I amphipathic helix. This phosphorylation event, likely by reducing membrane association and insertion, favors vesiculation. In contrast, non-phosphorylated form, through deeper membrane insertion, supports tubulation [12, 120, 121]. Accordingly, EndoA1 with phosphorylated Ser^75^ supports synaptic macroautophagy by promoting the formation of highly curved membranes, rather than participating in endocytosis (Fig. 2) [110, 122]. Phosphorylation by Rho-associated kinase ROCKII of EndoA1-Thr^14^ also inhibits endocytosis: it disrupts EndoA1 binding to the adaptor protein CIN85, causing reduced EGFR internalization [123, 124]. Localized within the H0 helix, Thr^14^ phosphorylation may also reduce insertion within the plasma membrane. Phosphorylation of EndoA2 by Src kinase on Tyr^315^, within its SH3 domain, alters dynamin binding and impairs endocytosis of the membrane-bound metalloproteinase MT1-MMP [125]. EndoA phosphorylation also occurs in other organisms, for example in ascidian *Ciona*, where phosphorylation of EndoA-Ser^263^ by dual specificity Tyr-phosphorylation-regulated kinase 1 (DYRK1) is required for optimal CME [126]. Finally, estrogen receptor-α (ERα), upon activation by 17β-estradiol, may also phosphorylate EndoA2, potentially affecting its endocytic functions [127].

EndoAs can also be regulated by the phosphorylation status of binding partners. For example, Cdk5- and GSK3β-mediated phosphorylation of dynamin and CRMP4 decreases their binding to EndoA2 and inhibits FEME [87]. Aside from phosphorylation, the E3 ubiquitin ligases Itch and Parkin bind to and ubiquitinate the SH3 domain of EndoA1 [128, 129], potentially regulating its stability and turnover. Moreover, EndoA2-Lys^294^, within the linker, was found conjugated to the ubiquitin-like protein MNSFβ, which regulates phagocytosis induced by the lectin receptor dectin-1 in macrophages [130, 131].

In addition to PTMs, calcium influx through voltage-gated Ca^2+^ channels also regulates EndoA functions in CM-SVE. This regulation appears to be mediated by Ca^2+^/calmodulin, which binds to both EndoA1 and EndoA2. Calmodulin bound to Ca^2+^ enhances EndoA1 association with the plasma membrane and the cytoskeletal regulator p140Cap [132], and promotes EndoA2-mediated membrane tubulation [133]. Alternatively, EndoAs may act as direct Ca^2+^ sensors, since Ca^2+^ initiates an EndoA1 conformational shift from a rigid, membrane-associated form to a more flexible cytosolic state that aids autophagosome formation (Fig. 2) [114]. Ca^2+^ is also suggested to bind directly to EndoAs, and its binding within the EndoA2 linker region induces an auto-inhibited conformation which blocks interactions with endocytic partners [45, 134]. The crystal structure of EndoA1 further suggests potential Ca^2+^ coordination at its N-BAR domain [8]. However, direct Ca^2+^ binding to EndoAs remains debated, as microcalorimetry failed to confirm such interactions [11]. Auto-inhibition via intramolecular interaction between the H0 helix and SH3 domain has also been proposed: such interaction would stabilize H0 helical folding in the cytosol and would be released upon membrane association [135]. An alternative mechanism rather suggests intradimer, intermonomer EndoA1 auto-inhibitory H0:SH3 interactions, relieved by SH3 ligand binding [136]. Finally, EndoAs are also likely transcriptionally regulated via mechanisms that remain mostly unexplored but which, based on dysregulated expression in various cancer types and other diseases (see below), may be implicated in pathogenesis.

## The EndoA proteins in physiology and pathology

### Neurodegeneration

With central roles in neuronal synaptic vesicle recycling, AMPAR trafficking, and autophagy (Fig. 2), EndoAs appear to be essential for synaptic homeostasis, and dysregulated EndoAs contribute to neurodegeneration. EndoA1 is overexpressed in both patients and mouse models of Alzheimer’s disease (AD) [137], Parkinson’s disease (PD) [138], and epilepsy [139]. Elevated EndoA1 promotes AD by augmenting Germinal Center Kinase-mediated activation of Jun N-terminal, leading to neuronal apoptosis [137], but also by increasing amyloid-β accumulation, which triggers synaptic dysfunction and cognitive decline [140, 141]. In PD, EndoA1 genetically and functionally interacts with the Parkinson-related proteins Parkin ubiquitin ligase [142] and LRRK2 kinase [143]. Mutated, hyperactive LRRK2 is commonly observed in PD patients [144], leading to EndoA1 hyperphosphorylation, consequently exacerbating its functions in synaptic autophagy and contributing to dopaminergic neuron degeneration [110, 122]. In contrast, *SH3GL2* mutants identified by genome-wide association studies increase PD risk by impairing EndoA1 functions in synaptic autophagy [114, 145, 146]. In epilepsy, EndoA1 overexpression influences the cell surface abundance of AMPARs, consequently favoring seizure susceptibility and activity [139]. Genetic studies also link *SH3GL2* polymorphisms and altered expression to schizophrenia [147, 148], suggesting broader neurological relevance. While EndoA3 remains less studied, it has been implicated in amyloid-β clearance in glioma cells [149] and in pathological aggregation of the Huntington disease exon 1 protein HDe1p [150]. These studies suggest that, among other mechanisms, EndoA functions in autophagy are critical to avoid pathological protein aggregation in neurons.

### Cardiovascular functions

Being ubiquitously expressed, EndoA2 has diverse functions in physiology and pathology, including in the cardiovascular system. In vascular endothelial cells, EndoA2 promotes VEGF-induced, FEME-mediated VEGFR2 internalization, and stimulates VEGFR2 autophosphorylation at Tyr^1214^. This activates downstream PAK and p38 kinases signaling from endosomes, promoting sprouting angiogenesis. Importantly, EndoA2 does not influence VEGFR2-Tyr^1175^ autophosphorylation, nor downstream ERK-driven proliferation, highlighting that distinct endocytic routes shape specific signaling and cellular outcomes [88]. In vascular smooth muscle cells, EndoA2 also regulates cell volume by coordinating trafficking to the plasma membrane of the chloride channel ClC-3 that facilitates Cl^−^ efflux upon cell swelling [151]. Interestingly, EndoA2 has cardiovascular protective effects in post-infarction cardiac injury and heart failure by preventing ER stress and subsequent apoptosis [152]. It similarly inhibits apoptosis in basilar artery smooth muscle cells and cardiomyocytes by binding to pro-apoptotic Bax, preventing its translocation into mitochondria [153], and by sustaining autophagy [113], respectively. EndoA2 functions in autophagy, combined with its regulation of angiotensin II type 1 receptor trafficking, further mitigate cardiac hypertrophy [154, 155]. EndoA2 also contributes to atherosclerosis, both by upregulating scavenger receptors expression and by promoting endocytosis of scavenger receptor-bound oxidized low-density lipoprotein deposits, promoting the conversion of macrophages into lipid-accumulating foam cells [156]. It further influences vasodilatation by inhibiting 17β-estradiol-induced Akt, ERK, and endothelial NOS activation [127]. In the brain, EndoA1 also downregulates EGFR/ERK signaling, which reduces the expression of the tight junction associated proteins Occludin and ZO-1, thereby increasing blood–brain barrier permeability [157].

### Other functions

In the kidney, EndoA2 exerts anti-fibrotic functions by binding to the type II TGF-β receptor, thereby preventing its interaction with the type I receptor and inhibiting downstream signaling [158]. EndoAs are also essential for kidney glomerular functions, as triple knockout mice show abnormal podocyte foot process formation and severe proteinuria [159]. In the peripheral nervous system, EndoA2 influences mechanosensation by promoting plasma membrane targeting of the mechanically-sensitive channel Piezo2 [160]. EndoA2 also has immune functions: in B-cells, it mediates antigen-bound BCR internalization and transferrin-mediated iron uptake, influencing antibody response and B-cell proliferation [86]. Consistently, a patient with mutated *SH3GL1* exhibits B-cell dysfunction and primary antibody deficiency [161]. EndoA2 also promotes T-cell receptor endocytosis and signaling, while its overexpression contributes to aberrant T-cell activation and autoimmune responses, as observed in rheumatoid arthritis [162].

Pathogens also exploit EndoAs for host entry: EndoA2 mediates the uptake of Shiga and Cholera toxins [89], *T. cruzi *[90], and enterovirus 71 [91], while the ALCAM-EndoA3 axis promotes internalization of human adenovirus species B [163]. EndoA2 may further influence pathogen morphogenesis, as suggested by its interactions with the viral proteins Cytomegalovirus pM50 [164] and murine leukemia virus Gag [165]. Though less characterized, EndoA2 expression in fat depots has been linked to obesity [166], and mutated EndoA2 is associated with scoliosis [167]. While future *in vivo* studies are required for a more comprehensive understanding of EndoA implications in the mechanisms of pathologies, these observations collectively indicate that they are potential therapeutic targets and/or biomarkers in selected diseases.

## Focus on EndoAs in the pathophysiology of cancer

The critical contribution of endocytosis to cancer, by regulating the cell surface turnover of signaling, adhesion, and immune proteins, as well as by influencing therapeutic drug delivery, no longer needs to be demonstrated [168]. It is therefore not surprising that endocytic proteins are frequently dysregulated in cancer, and EndoAs are no exception. Of course, EndoA implications in various other cellular processes may further contribute to cancer pathogenesis. Large systematic screens have revealed aberrant EndoA expression, post-translational modifications, and mutations in multiple cancer types (https://bioportal.bioontology.org/). More targeted investigations have confirmed the contribution of dysregulated EndoAs to cancer progression, primarily through altered receptor-mediated signal transduction, ultimately impacting cancer cell stemness, migration, invasion, proliferation, and drug resistance. While, to the best of our knowledge, EndoA2 is primarily reported to be pro-tumoral, both pro- and anti-tumoral contributions have been reported for EndoA1 and EndoA3 (Table 1).

### EndoA1 (***SH3GL2***)

Compared to healthy tissues, *SH3GL2* expression is decreased in different brain cancer types, including glioblastoma [169, 170], neuroblastoma [171], and pilocytic astrocytoma [172]. Interestingly, *SH3GL2* expression was also detected in healthy bladder [173], breast [174], vulvar [175], laryngeal [176], and lung [177] tissues, again with a decreased expression in the corresponding urothelial [173], breast [174, 178], vulvar squamous cell [175], head and neck squamous cell [179, 180], and non-small-cell lung [177, 181] carcinoma tissues. Several mechanisms have been reported to decrease *SH3GL2* expression in tumor cells, including chromosomal deletion [173, 174, 181], promoter hypermethylation [174, 175, 179, 180], microRNA upregulation [170], and single nucleotide polymorphisms affecting transcript stability [182]. Reduced abundance/functionality may also occur at the protein level, as exemplified by a frameshift mutation within the EndoA1 SH3 domain in head and neck dysplastic lesions and squamous cell carcinomas [179]. Consistent with decreased tumoral expression, EndoA1 is mostly reported to be a tumor suppressor. Indeed, reduced *SH3GL2* expression correlates with increased tumor grade and invasion in urothelial carcinoma [173], and with higher tumor malignancy in vulvar squamous cell carcinoma tissues [175]. Similarly, reduced *SH3GL2* expression in retinoblastoma cells, particularly observed in invasive cells, augments migration and tumor growth and, by increasing the amount of myeloid-derived suppressor cells, promotes immunosuppression [183]. Further suggesting tumor suppressor features, exogenous *SH3GL2* expression in urothelial carcinoma, lung adenocarcinoma, and glioma cells decreases their proliferation and migration [169, 173, 177].

**Fig. 4:**
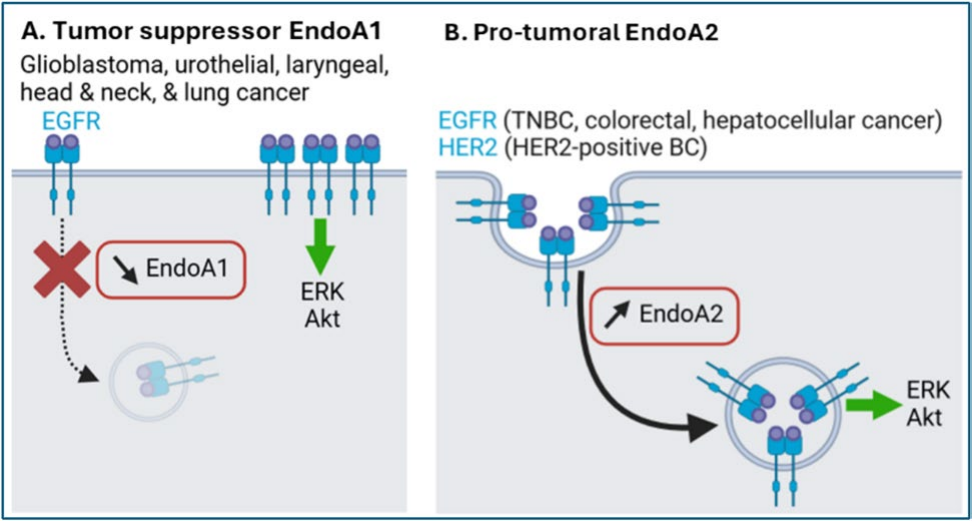
EndoA1 downregulation and EndoA2 upregulation lead to oncogenic signaling in selected cancer types. **A** EndoA1 downregulation observed in glioblastoma, urothelial, laryngeal, head and neck, and lung cancer leads to reduced endocytosis of activated EGFR, causing its accumulation at the cell surface and hyperactive signal transduction to ERK and Akt. **B** EndoA2 upregulation in triple-negative (TN) breast cancer (BC), HER2 BC, colorectal, and hepatocellular cancer promotes endocytosis of ligand-activated EGFR/HER2 receptors, likely by FEME. This increased internalization augments endosomal signal transduction to ERK and Akt. Generated with BioRender.

Mechanistically, the oncogenic implications of lowered *SH3GL2* expression have mostly been attributed to reduced EGFR endocytosis, leading to hyperactive signal transduction from the plasma membrane (Fig. 4A). For example, urothelial and laryngeal carcinoma cell lines display reduced EGFR internalization and increased ERK signaling [173, 184]. Increased ERK, but also Akt signaling, is also observed in *SH3GL2* low-expressing glioblastoma stem cells, which correlates with increased proliferation, migration, and invasion [170]. Similarly, low *SH3GL2* expression in dysplastic and squamous cell head and neck carcinoma samples correlates with higher levels of activated EGFR, as well as with poor prognosis [180]. Oppositely, exogenous EndoA1 expression in non-small cell lung cancer cells reduces EGFR activation and decreases proliferation [181]. Together, these studies support that EndoA1 is involved in EGFR endocytosis, possibly through clathrin-/CIN85 adaptor-/Cbl ubiquitin ligase-dependent mechanism [69] or CIE [185], leading to decreased signal transduction (Fig. 4A). In glioma cells, EndoA1 further downregulates STAT3 which, in turn, decreases the expression of matrix metalloproteinase-2 to limit cell migration and invasion [169], suggesting implications in additional signaling pathways. Curiously, EndoA1 tumor suppressor activities in breast cancer cells were also attributed to its translocation to the mitochondria where, by mediating the release of superoxide and cytochrome C, it would induce apoptosis to halt tumor growth and metastasis [178]*.* In contrast, a few other studies suggest that *SH3GL2* is overexpressed in gastric [186] and esophageal cancer samples [187] compared to healthy counterparts [188]. It is similarly overexpressed in pediatric medulloblastoma and ganglioglioma [189], where it promotes HGF-induced growth and invasiveness of medulloblastoma cells [190], possibly due to increased HGFR endocytosis and signaling from endosomes. However, future studies are required to clearly establish and understand possible EndoA1 pro-tumoral activities.

### EndoA2 (***SH3GL1***)

EndoA2 is predominantly characterized as pro-tumorigenic. It is overexpressed in both HER2-positive and triple-negative breast cancer, which correlates with poor prognosis [191, 192]. In HER2-positive cells, EndoA2 promotes HER2 endocytosis induced by EGF and by the therapeutic antibody trastuzumab, leading to endosomal signal transduction to ERK and Akt, ultimately stimulating migration and invasion [191]. Similarly, EndoA2 in triple-negative breast cancer cells mediates EGFR endocytosis and signaling to ERK/Akt, sustaining motility and invasiveness. EndoA2 pro-tumoral activity was confirmed *in vivo*, as it promotes triple-negative breast cancer growth and metastasis in xenograft mice [192]. These studies highlight that, in contrast to EndoA1 that decreases EGFR signaling at the plasma membrane, EndoA2-mediated internalization of ligand-stimulated receptors (likely by FEME) promotes oncogenic endosomal signaling (Fig. 4B). Moreover, phosphorylation of EndoA2-Tyr^315^ by Src, which inhibits MT1-MMP endocytosis and increases ECM degradation [125], also accelerates tumor progression in a murine breast cancer model by promoting epithelial-mesenchymal transition and mammary cancer stemness [193].

*SH3GL1* is pro-tumoral in other cancer types: it is overexpressed in osteosarcoma [194], hepatocarcinoma [195], and diffuse large B-cell lymphoma tissues [196], where it positively correlates with poor prognosis. In osteosarcoma cells, EndoA2 supports interleukin-6 and VEGF-stimulated phosphorylation of p130^cas^, Akt, FAK, and GSK-3β proteins, which promotes cell cycle and migration [194]. In liver cancer cells, EndoA2 binds to and activates β-catenin, driving cell proliferation and stemness [195]. In diffuse large B-cell lymphoma cells, it promotes cell survival and chemoresistance by inhibiting ferritin-mediated ferroptosis [196]. EndoA2 also confers chemoresistance in colorectal cancer cells by stimulating EGFR/ERK signaling (Fig. 4B), which activates the transcription factor AP-1 that increases P-glycoprotein drug efflux pump expression [197]. Interestingly, *SH3GL1* overexpression has been linked to downregulation of tumor-suppressive microRNAs: in medulloblastoma cells, reduced miR-218 increases *SH3GL1* expression, which stimulates ERK activation and cell proliferation [198]. Similarly, downregulation of miR-3663-3p in hepatocellular carcinoma cells elevates *SH3GL1* expression, correlating with hyperactive EGFR/ERK/NF-κB signaling [199].

Interestingly, in acute myeloid leukemia, *SH3GL1* is found fused to the mixed-lineage leukemia (*MLL*) gene, which encodes a histone methyltransferase essential for Hox gene regulation during development and hematopoiesis [200]. The resulting MLL-EEN fusion protein promotes myeloid progenitor proliferation and induces leukemia in mice [201]. Strikingly, MLL-EEN localizes to the nucleus where it is hypothesized to function as a transcriptional activator [202]. By binding EndoA2, MLL-EEN also relocalizes both EndoA2 [202] and its binding partner EEN-binding protein [203] to the nucleus. As cytosolic EndoA2 and EBP suppress Ras signaling, their nuclear sequestration limits this effect, increasing Ras-induced cell transformation [203]. In mice, MLL-EEN also impairs anti-leukemic immune response by disrupting myeloid dendritic cell differentiation [204]. Interestingly, the leukemia-associated AML1-ETO fusion gene upregulates *SH3GL1* expression, further contributing to leukemogenesis by promoting proliferation and myeloid transformation [205]. Collectively, these studies demonstrate the wide spectrum of EndoA2 pro-tumoral implications across multiple cancer types, but with connections to its membrane remodeling functions that remain sometimes elusive.

### EndoA3 (***SH3GL3***)

Like *SH3GL1, SH3GL3* expression is lower in glioblastoma compared to healthy brain tissues, with further reduction in higher-grade gliomas, suggesting tumor- suppressive roles [206]. Reduced expression is attributed to downregulation of the RNA binding protein ELAVL2, limiting stabilization of *SH3GL3* transcripts [207]. Functionally, EndoA3 downregulates STAT3 expression and activation in glioblastoma cells, inhibiting oncogenic stemness, proliferation, and migration [206]. However, *SH3GL3* expression is reported to be upregulated in glioma infiltration zones, as well as in non-functioning pituitary adenomas [208]. Moreover, it promotes matrix metalloproteinase activity in glioma cells, which supports invasiveness [209]. These findings suggest complex EndoA3 contributions to brain cancer, which seem to be type- and grade-dependent.

Compared to healthy counterparts, *SH3GL3* expression is also reduced in lung cancer tissues [210], where upregulation of the long noncoding mRNA MIR210HG leads to the recruitment of the DNA methyltransferase DNMT1 to the *SH3GL3* promoter, inhibiting its transcription [211]. In lung cancer cells, EndoA3 has tumor-suppressive roles by inhibiting proliferation and migration, inducing apoptosis, and, via upregulation of the cyclin-dependent kinase inhibitor p21, arresting cell cycle [210, 211]. Similarly, lower *SH3GL3* expression in metastatic versus non-metastatic oral squamous cell carcinoma supports tumor suppressive roles [212]. Conversely, *SH3GL3* overexpression is detected in multiple myeloma [213], colon cancer [214], and melanoma [215] specimens, where it promotes tumorigenesis. In CD138-negative multiple myeloma clonogenic cells, high *SH3GL3* expression activates PI3K and FAK kinases to enhance migration and invasion, promote stemness, and increase the expression of multidrug resistance markers leading to chemoresistance [213]. In colon cancer, *SH3GL3* expression increases in higher-grade tumors, and EndoA3 supports two pro-tumoral mechanisms: *(i)* cytosolic EndoA3 binds to the GEF Tiam1, activating Rac1 and subsequent cell migration, and *(ii)* membrane-associated EndoA3 promotes proliferation via its endocytic activity [214]. More indirectly, metastasis-associated protein 1 (MTA1) binds to EndoA3 which, by regulating its endocytic functions, potentially contributes to MTA1 oncogenicity [216]. Interestingly, EndoA3-operated CIE of Ig-like CAMs may also influence cancer cell adhesion and migration. Supporting this, ALCAM endocytosis by EndoA3 reduces adhesion and promotes migration of osteosarcoma cells [84], suggesting implications in metastasis. However, EndoA3-mediated endocytosis also supports anti-tumor immunity: internalized ALCAM and ICAM1 in cancer cells undergo retrograde trafficking and polarized redistribution to the plasma membrane, where they contribute to the formation of immune synapses with CD8^+^ T-cells, promoting their activation [103]. Together, these studies underscore complex contributions of EndoA3 as a tumor suppressor or pro-tumoral in various cancer types, as highlighted by EndoA3 supporting both cancer cell migration and anti-cancer immune response in the same cellular context, warranting further investigation.

## The EndoA proteins: possible clinical applications?

The involvement of EndoAs in diverse pathologies highlights their potential for clinical applications. First, the aberrant EndoA expression observed in neurodegenerative, cardiovascular, and cancer contexts suggests possible uses as biomarkers. Since EndoA2 overexpression elicits high production of anti-EndoA2 autoantibodies in patients with low-grade glioma, breast, liver, gastric, and colon cancer [217, 218], serum autoantibody dosage could also be used for minimally-invasive early diagnostic. Given their pro-tumoral and neurodegenerative actions, as well as their implications in pathogen infection, EndoAs also represent promising therapeutic targets. The absence of overt phenotype in single EndoA knockout mice further supports the clinical relevance of selective therapeutic inhibition. Strategies may include trapping with therapeutic antibodies, interference using therapeutic peptides, or inhibition with small molecule inhibitors. Alternatively, the identification of miRNAs targeting the *SH3GL2/1/3* genes, as well as the use of therapeutic siRNAs, also provides possibilities to artificially mitigate pathological EndoA overexpression. Conversely, enhancing *SH3GL2/1/3* gene expression may be beneficial in diseases where they exert protective effects, for example as tumor suppressor in some cancer types or in cardiovascular diseases. Oligonucleotide therapies using small-activating RNAs or synthetic mRNAs could for example be explored. Therapeutic recombinant proteins are another alternative: for example, EndoA2 fused to the cell-penetrating peptide Tat penetrates the blood–brain barrier in gerbils, preventing ischemia-induced hyperactivity and oxidative stress [219]. Despite these prospects, no EndoA-targeted therapy currently exists, reminding us that their design and development are far from trivial. Among others, EndoA high structural similarity complicates selective targeting, as required to minimize possible side-effects. Less direct therapeutic approaches may also target upstream regulators, such as kinases, or even EndoA cargoes, but again with the possibility of deleterious off-targets. Finally, EndoA-mediated endocytosis could be exploited for targeted drug delivery. For example, immunoliposomes coupled with anti-ALCAM single-chain antibody fragments (scFv) have been used to deliver anti-tumor agents to prostate cancer cells [220], opening avenues for the future design of functionalized nanotherapeutics targeting EndoA-mediated endocytic axes.

## Are EndoAs same same but different?

Human EndoAs share approximately 65% amino acid sequence identity, along with conserved domain organization (Fig. 1). While EndoA1-3 triple knockout mice undergo perinatal mortality, single knockouts show no overt phenotype, implying partial redundancy [26]. However, it has become clear over the last decade that EndoAs also have distinct, paralog-specific functions. For instance, although EndoA3 is expressed in the brain, it does not appear to participate in synaptic vesicle endocytosis like EndoA1 and -A2 [46]. Additionally, EndoAs at the plasma membrane show non-overlapping localization profiles [84], suggesting affinity for different local membrane domains and a low probability of EndoA heteromerization in cells. Importantly, the EndoA proteins mediate distinct CIE modalities: EndoA2 facilitates FEME of ligand-activated receptors [83], while EndoA3 mediates the internalization of Ig-like CAMs [84]. However, the extent to which EndoA functions are conserved or divergent remains poorly defined, due to several limitations: *(i)* many studies focus on a single EndoA homolog, without assessing functional overlap with the others, *(ii)* some EndoA functions were characterized in model organisms such as *D. melanogaster* and *C. elegans* which express only one EndoA protein, and *(iii)* some studies refer generically to EndoAs without specifying the homolog being examined.

A key open question is how the three highly similar EndoAs may have partially distinct functions. One possibility could be EndoA-specific regulation by PTMs, as supported by the identification of phosphorylation sites on residues non conserved across the three human EndoAs in high-throughput phosphoproteomic screens (Fig. 1C). Functional differences may also arise from distinct protein:protein interactions. For example, EBP binds to EndoA2 and -A3, but not to EndoA1 [203], and the protein:protein interaction database BioGRID reports both shared and unique EndoA interactors. Specific interactions with cargoes and adaptor proteins, but also with proteins commonly involved in endocytosis like GTPases and molecular motors, are of particular interest to understand their involvement in different CIE modalities. Importantly, such differences in PTMs and binding partners may also influence EndoA subcellular localization, in particular their shuttling from the cytosol to cellular membranes, thereby possibly resulting in different kinetics at the cell surface. Variations in preferential lipid binding could also induce membrane partitioning, resulting in different functions. Interestingly, the EndoA H0 amphipathic helices show different net charge and hydrophobicity, possibly influencing lipid specificity and binding to specific local membrane domains [221]. However, the lipid-binding preferences of the different EndoA proteins remain to be further explored. Small variations in EndoA 3D structure may also result in membrane selectivity, for example towards specific curvature. Together, these open questions highlight the importance of structure–function studies comparing the three human EndoAs to understand the scope of individual functions at the cellular and pathophysiological levels.

## Discussion and prospects

This review illustrates pivotal functions of the EndoA proteins, primarily in endocytosis. In particular, recent advances highlight that EndoAs behave as a versatile module interacting with various other proteins to form functional endocytic machineries that are selectively recruited in several modalities. Interestingly, EndoAs are involved both in rapid (UFE, FEME) and slower (CME) endocytic mechanisms. *In vitro* kinetic studies indicate that EndoAs bind to biological membranes as dimers, then oligomerize into helical lattices on the membrane to induce tubulation [222]. These studies further reveal that EndoAs first act as curvature sensors, and require 30 ms to transition to membrane benders. While EndoAs induce local curvature within approximately 100 ms, larger membrane deformations like tubulation occur on a sec to min timescale. These studies highlight that the duration of endocytic events likely constrains the mode of action of EndoAs [222, 223].

Importantly, key questions remain unanswered in the EndoA field. First, the molecular mechanisms regulating such dynamic recruitment in selective endocytic routes are still poorly understood. Systematic identification of EndoA cargoes is also needed to fully appreciate the scope of their cellular functions. Complicating the study of EndoA-mediated endocytosis, cargoes are often internalized via multiple modalities, depending on the cellular context. Similarly, most endocytic players are shared among various modalities, with regulated spatial and temporal recruitment allowing more cellular plasticity. While complex and mostly overlooked, such crosstalk, compensation, and competition between EndoA-mediated and other endocytic modalities must be addressed. Beyond cargo internalization, downstream consequences – such as impact on signaling, adhesion, or immunity – although sometimes investigated, also warrants future attention. Emerging evidence for EndoA involvement in additional cellular processes significantly expands their functional repertoire. Yet, the molecular determinants governing context-specific EndoA functions at given subcellular localizations (for example Ca^2+^ signaling and phosphorylation regulating EndoA functions in endocytosis versus autophagy) should be further studied. Unfortunately, fully answering those open questions is hindered by technical challenges. First, their high similarity limits the availability of EndoA-specific molecular tools, particularly antibodies. Consequently, many studies still rely on ectopic expression of tagged proteins prone to artifacts. Similarly, the development of isoform-specific small molecule inhibitors would greatly facilitate functional and mechanistic studies, complementing siRNA-based approaches. Adding more complexity, inconsistent nomenclature, combined with the lack of isoform specificity in some earlier studies, complicate the integration of past findings and needs to be addressed in future studies.

Many studies link EndoAs to diseases, reminding us of the intricate connection between membrane trafficking and pathologies. However, limitations persist: causal relationships are often unconfirmed, most underlying mechanisms remain incompletely characterized, and available data are frequently restricted to *in cellulo* systems. More generally, despite established single- and triple-knockout mouse models [26], *in vivo* analyses remain limited, restricting our understanding of EndoA functions in health and disease. Nevertheless, and as discussed above, existing studies collectively support the potential for EndoA-based diagnostic and therapeutic strategies. In conclusion, we anticipate that future research on the EndoA proteins will continue to shape their mechanistic understanding and functional landscape, while suggesting promising translational applications.

## Data Availability

This manuscript is a review article and does not include any data nor materials.
